# Prevalence, Risk Factors, and Complications of Cholelithiasis in Adults With Short Bowel Syndrome: A Longitudinal Cohort Study

**DOI:** 10.3389/fnut.2021.762240

**Published:** 2021-11-29

**Authors:** Xuejin Gao, Li Zhang, Siwen Wang, Yaqin Xiao, Deshuai Song, Da Zhou, Xinying Wang

**Affiliations:** ^1^Department of General Surgery, Jinling Hospital, Medical School of Nanjing University, Nanjing, China; ^2^Department of General Surgery, Jinling Hospital, Southern Medical University, Guangzhou, China; ^3^Department of General Surgery, Jinling Hospital, Nanjing Medical University, Nanjing, China

**Keywords:** cholelithiasis, short bowel syndrome, prevalence, risk factors, clinical consequence

## Abstract

**Background:** Patients with short bowel syndrome (SBS) are at a high risk of cholestasis or cholelithiasis. This study aimed to determine the incidence, risk factors, and clinical consequences of cholelithiasis in adults with SBS over an extended period.

**Methods:** All eligible adults diagnosed with SBS and admitted to a tertiary hospital center between January 2010 and December 2019 were retrospectively identified from the hospital records database. Kaplan–Meier analysis was used to estimate the cumulative incidence of SBS during the 10-year period. For assessment the risk factors for cholelithiasis, we used multivariate Cox proportional hazards model with estimation of hazard ratio (HR) with 95% confidence intervals (95 %CI).

**Results:** This study enrolled 345 eligible patients with SBS. Kaplan–Meier analysis revealed that 72 patients (20.9%) developed cholelithiasis during the 10-year observation period. In multivariate analyses using the Cox proportional hazard model revealed that the remnant jejunum (HR = 2.163; 95% confidence interval [CI]: 1.156–4.047, *p* = 0.016) and parenteral nutrition dependence (HR = 1.783; 95% CI: 1.077–2.952, *p* = 0.025) were independent risk factors for cholelithiasis in adults with SBS. Twenty-eight patients developed symptoms and/or complications in the cholelithiasis group. Proportions of acute cholecystitis or cholangitis and acute pancreatitis were significantly increased in the cholelithiasis group compared with the non-cholelithiasis group (31.9 vs. 7.7%, *p* < 0.01; and 6.9 vs. 1.1%, *p* = 0.003, respectively).

**Conclusion:** Because of the adverse clinical consequences of cholelithiasis, adult patients with SBS should be closely monitored, and preventive interventions should be considered.

**Clinical Trial Registration:** www.ClinicalTrials.gov, identifier: NCT04867538.

## Background

The estimated overall prevalence of cholelithiasis is 10–15% in the general population, with some differences across countries and the majority of patients being asymptomatic (1, 2). However, it has been demonstrated that patients with short bowel syndrome (SBS) are at high risk of developing cholelithiasis due to altered gastrointestinal anatomy and physiology (3–5). SBS is a rare disease, defined as loss of bowel mass from extensive resection of the small intestine (6–8). Depending on the severity of malabsorption, it will lead to intestinal failure (IF), defined as the reduction of gut function below the minimum necessary for the absorption of macronutrients and/or water and electrolytes, resulting in a situation where intravenous supplementation is required (9). The incidence of SBS is about 2 per million per year, and its prevalence is about 20 per million in Europe and the United States (8, 10); however, the exact epidemiology is unknown (10).

Several previous reports have demonstrated an increased incidence of gallbladder disease in patients with SBS on long-term parenteral nutrition (PN) (3, 4). In addition to biliary sludge, patients develop cholelithiasis and symptomatic biliary disease (3). The prevalence of biliary sludge in patients on long-term PN is as high as 100% and is a predisposing factor for cholelithiasis (3, 11). However, the disappearance of sludge with the resumption of oral intake has been noted, and not all patients progress to cholelithiasis. The precise pathophysiologic processes that perpetuate gallbladder sludge and lead to cholelithiasis formation are largely unknown, but gallbladder stasis plays a key role (3, 12).

Decreased oral intake or the lack enteral feeding and the use of long-term PN predispose patients to sludge and cholelithiasis formation by promoting stasis in the biliary tree (13). A previous study reported that 25–38% of patients receiving long-term PN developed symptomatic or asymptomatic cholelithiasis (4, 14). The fact that the use of long-term PN predisposes patients to cholelithiasis formation was not surprising. However, only limited data are available regarding cholelithiasis in patients with SBS. The incidence of cholelithiasis in patients with SBS has only been reported in a few small sample studies (4, 5, 15). Furthermore, few studies have focused on identifying the risk factors and clinical consequences of cholelithiasis in patients with SBS. There have been significant improvements in medical health care and PN support in the 21st century. At present, the incidence and risk factors for cholelithiasis in adult patients with SBS remain unclear.

Therefore, this study aimed to provide comprehensive and long-term data on the incidence of cholelithiasis in a cohort of patients with SBS to identify the prevalence, risk factors, and clinical consequences of cholelithiasis in adult patients with SBS to aid in the development of protective interventions.

## Materials and Methods

### Ethics Statements

The study was conducted in accordance with the principles of the Declaration of Helsinki, and the study protocol was approved by the ethics committee of Jinling Hospital (Identifier: 2020ZFYJ-014-05) and registered at ClinicalTrials.gov (Identifier: NCT04867538). Because of the retrospective nature of the study, patient consent for inclusion was waived.

### Study Design and Participants

This was a retrospective cohort study of a prospectively maintained audit database of all patients with SBS managed at Jinling Hospital. All adults with SBS admitted to Jinling Hospital's Intestinal Failure Clinical Nutrition Center from January 2010 to December 2019 were retrospectively identified from the hospital records database included in this study. The prospective database was maintained to guide nutrition support therapy for patients with chronic intestinal failure (CIF) (including oral diet, parenteral/enteral nutrition, and home parenteral/enteral nutrition). Our Intestinal Failure Clinical Nutrition Center established a special nutrition support team (an interdisciplinary team composed of gastroenterologists, surgeons, pharmacists, clinical dietitians, and nurses) that is responsible for developing nutrition support programs and plans and performing long-term follow-up after discharge of each patient with IF associated with SBS.

The inclusion criteria were as follows: adults with SBS and an expected survival time of more than 6 months. SBS was defined as intestinal malabsorption disorder caused by extensive bowel resection with a remnant small intestine length <200 cm (9, 10). The diagnosis of SBS was made in our Intestinal Failure Clinical Nutrition Center by two gastroenterologists, three surgeons, and one dietitian (16, 17), and the remnant small intestine length was determined according to the patient's surgical record or gastrointestinal barium examination. The exclusion criteria were as follows: age <18 years, patients with cholecystectomy, patients with a history of cholelithiasis before SBS diagnosis, patients with biliary tract obstruction, and patient with no abdominal imaging (computed tomography [CT], magnetic resonance imaging [MRI], or ultrasonography) after SBS diagnosis.

### Demographic and Clinical Variables

Patient demographic data at admission, including age, sex, height, body weight, the body mass index (BMI), presence of diabetes mellitus, presence of hypertension, nutritional risk screening 2002 (NRS-2002) score, patient-generated subjective global assessment (PG-SGA) grade, and serum concentrations of albumin, prealbumin, transferrin, retinol-binding protein, insulin-like growth factor-1, total bilirubin, direct bilirubin, alanine aminotransferase, aspartate aminotransferase, alkaline phosphatase, gamma glutamyltransferase, blood urea nitrogen, hemoglobin, and platelets, were collected from the database. PN dependence, primary disease, and intestinal anatomy (including the type of anatomy, remaining length of the small intestine, type of remaining small intestine [mainly the remnant jejunum or ileum], and the presence of an intact ileocecal valve, colon-in-continuity, and colon integrity) were also recorded.

According to anatomical criteria, SBS can be classified into three types: type I: end-jejunostomy (patients undergoing end-jejunostomy have the ileum and colon completely removed, while a portion of the jejunum is retained and forms the end of the intestine) (18); type II: jejuno-colonic anastomosis (patients with type II SBS have a jejuno-ileal resection, in which all or most of the ileum is removed with preservation of the colon and jejuno-colonic anastomosis formation) (18); and type III: jejuno-ileal anastomosis (this type is represented predominantly by jejunal resection leaving ≥10 cm of the terminal ileum and the entire colon intact) (9, 18). The duration of SBS was defined as the time interval between SBS diagnosis and the last hospital visit (16, 17).

### Diagnosis of Cholelithiasis

At admission, patients with SBS were asked about their history of cholelithiasis. The diagnosis of cholelithiasis at any point during the period of SBS follow-up was recorded, along with the modality and indication for investigation that resulted in the diagnosis (symptoms suggestive of cholelithiasis, abnormal liver function test results, or incidental findings) (15). Cholelithiasis was diagnosed based on history taking, the physical examination, blood examination, and abdominal imaging (ultrasonography, abdominal CT, and MRI).

Patients with SBS were divided into two groups: cholelithiasis and non-cholelithiasis. Patients were classified into the cholelithiasis group if they had been definitively diagnosed with cholelithiasis after SBS diagnosis, or if symptomatic or asymptomatic cholelithiasis was confirmed on abdominal imaging (CT, MRI, or ultrasonography). The symptoms, types of complications of cholelithiasis, and their consequent management were also recorded. All patients were followed-up at the Intestinal Failure Clinical Nutrition Center's outpatient clinic at Jinling Hospital at least every 6 months during the study period.

### Statistical Analysis

Numerical data are presented as mean ± standard deviation or median (range). Categorical variables are expressed as number and percentage. We used the Kolmogorov-Smirnov test to assess whether continuous data were normally distributed. We performed group comparisons using the chi-square test or Fisher exact tests for categorical variables, and the Student *t*-test or Mann–Whitney U-test for continuous variables when appropriate. A Kaplan-Meier curve was generated for the incidence of cholelithiasis (15). The Cox proportional hazard multivariate modeling was used to evaluate potential risk factors associated with cholelithiasis in adults with short bowel syndrome. Results are presented as hazard ratio (HR) with 95% confidence intervals (95% CI). All tests were two-sided. Statistical significance was set at *p* < 0.05. All analyses were performed using SPSS (version 21.0; IBM Corp., Armonk, NY, USA).

## Results

### Demographic and Clinical Characteristics of the Study Population

**Figure 1:**
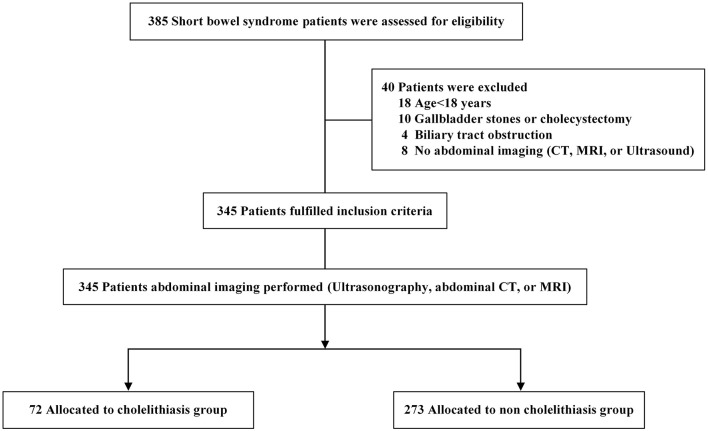
Flow diagram of patients with short bowel syndrome included in the study.

As depicted in Figure 1, 385 patients with SBS were recruited between January 2010 and December 2019. Forty patients were excluded for different reasons: 18 patients were minors, 10 had gallbladder stones or cholecystectomy, four had biliary tract obstruction, and 8 had no abdominal imaging (CT, MRI, or ultrasonography). Thus, among 345 patients with SBS who fulfilled the inclusion criteria, 72 were allocated to the cholelithiasis group and 273 were allocated to the non-cholelithiasis group (Figure 1 and Supplementary Figure 1).

The patients' mean age was 50.0 ± 16.2 years, and 236 (68.4%) were male. The median BMI was 18.2 (range, 16.4–21.2 kg/m^2^), median NRS-2002 score was 4.0 (range, 3.0–5.0), and PG-SGA grades were as follows: grade A in 36 (10.4%) patients, grade B in 133 (38.6%), and grade C in 176 (51.0%). Thirteen (3.8%) patients had type 2 diabetes mellitus, and 47 (13.6%) were hypertensive. The median duration of SBS was 36 months (range, 16–72 months). Additionally, 117 (34.0%) patients required PN at admission, while 70 (20.3%) required home PN (HPN) after hospital discharge. Baseline characteristics of the study population are shown in Table 1.

**Table 1:** Demographic and laboratory tests for the study population.

**Characteristic**	**All**	**Cholelithiasis**	**Non cholelithiasis**	***p* value**
**Number of patients (%)**	345	72 (20.9)	273 (79.1)	NA
Age (years)	51.0 (39.0–61.0)	50.0 (39.3–61.8)	51.0 (38.0–61.0)	0.691
Sex				**0.039**
Female	109 (31.6)	30 (41.7)	79 (28.9)	
Male	236 (68.4)	42 (58.3)	194 (71.1)	
Height (cm)	168 (162–172)	167 (162–170)	168 (162–172)	0.185
Body weight (kg)	51.0 (45.0–60.0)	49.5 (43.5–59.0)	52.0 (45.0–60.0)	0.208
BMI (kg/m^2^)	18.2 (16.4–21.2)	17.5 (16.2–21.3)	18.4 (16.5–21.1)	0.330
Diabetes mellitus (%)	13 (3.8)	2 (2.8)	11 (4.0)	0.620
Hypertension (%)	47 (13.6)	11 (15.3)	36 (13.2)	0.645
PG-SGA				**0.038**
A	36 (10.4)	4 (5.6)	32 (11.7)	
B	133 (38.6)	22 (30.6)	111 (40.7)	
C	176 (51.0)	46 (63.9)	130 (47.6)	
NRS2002	4.0 (3.0–5.0)	4.0 (3.3–5.0)	4.0 (3.0–5.0)	0.524
PN dependence (%)				**0.007**
Yes	116 (33.7)	34 (47.2)	82 (30.1)	
No	228 (66.3)	38 (52.7)	190 (69.9)	
Duration of SBS (months)	36 (16–72)	37 (17–74)	36 (15–71)	0.235
**Laboratory tests**				
Albumin (g/L)	35.5 ± 7.1	36.7 ± 6.5	35.2 ± 7.2	0.093
Prealbumin (mg/L)	213 (144–283)	216 (154–292)	212 (134–273)	0.539
Transferrin (g/L)	2.1 ± 0.77	2.1 ± 0.76	2.1 ± 0.78	0.986
RBP (mg/L)	34.5 (23.0–45.8)	34.0 (24.0–45.0)	35.0 (22.0–46.5)	0.813
IGF-1 (ug/L)	127 (70–173)	144 (107–231)	114 (69–158)	0.150
TBIL (μmol/L)	17.1 (10.6–28.2)	17.8 (12.2–33.6)	16.4 (10.1–27.2)	0.120
DBIL (μmol/L)	7.2 (3.6–13.2)	8.5 (4.5–18.8)	6.7 (3.4–12.7)	0.109
ALT (U/L)	33.0 (20.0–59.8)	34.0 (18.3–61.0)	33.0 (20.3–59.0)	0.994
AST (U/L)	28.0 (18.0–50.5)	27.5 (18.0–51.3)	28.0 (18.0–50.5)	0.953
ALP (U/L)	99 (69–161)	109 (74–194)	97 (69–156)	0.281
γ-GT (U/L)	49 (20–126)	56 (20–147)	48 (20–116)	0.580
Triglyceride (mmol/L)	1.22 (0.80–1.90)	1.17 (0.78–1.88)	1.25 (0.82–1.90)	0.602
Cholesterol (mmol/L)	2.78 (2.14–3.59)	2.97 (2.19–4.03)	2.71 (2.11–3.47)	0.144
BUN (mmol/L)	5.8 (4.0–8.1)	5.7 (4.1–9.1)	5.8 (3.9–8.0)	0.379
Serum creatinine (mmol/L)	57.0 (44.0–78.4)	60.4 (43.2–96.7)	56.1 (44.0–77.0)	0.272
Hemoglobin (g/L)	107.4 ± 24.7	105.8 ± 22.2	107.9 ± 25.3	0.516
PLT (x10^9^/L)	186 (131–235)	164 (129–227)	192 (132–238)	0.140

*Values were presented as n (%), or mean ± SD, or median (first-to-third interquartile range)*.

*P < 0.05 is indicated by black bold*.

*SBS, short bowel syndrome; BMI, body mass index; PG-SGA, Patient-Generated Subjective Global Assessment; NRS-2002, nutritional risk screening 2002; PN, parenteral nutrition; PLT, platelet; RBP, retinol-binding protein; IGF-1, insulin-like growth factor 1; TBIL, total bilirubin; DBIL, direct bilirubin; ALT, alanine aminotransferase; AST, aspartate aminotransferase; ALP, alkaline phosphatase; γ-GT, Gamma glutamyltransferase; BUN, blood urea nitrogen*.

The most common underlying diseases were mesenteric ischemia (*n* = 122, 35.4%), surgical complications (*n* = 109, 31.6%), volvulus (*n* = 53, 15.4%), trauma (*n* = 27, 7.8%), radiation enteritis (*n* = 21, 6.1%), Crohn disease (*n* = 8, 2.3%), and others (*n* = 5, 1.4%). Regardless of the cholelithiasis and non-cholelithiasis groups, the most common causes of SBS were mesenteric ischemia, surgical complications, and volvulus (Table 2 and Supplementary Figures 2A–C).

**Table 2:** The underlying diseases in adult patients with SBS with and without cholelithiasis.

**Variables**	**All**	**Cholelithiasis**	**Non cholelithiasis**	***p* value**
**Number of patients**	345	72	273	NA
The etiology of SBS (%)				0.392
Mesenteric ischemia	122 (35.4)	23 (31.9)	99 (36.3)	
Surgical complications	109 (31.6)	18 (25.0)	91 (33.3)	
Volvulus	53 (15.4)	14 (19.4)	39 (14.3)	
Trauma	27 (7.8)	8 (11.1)	19 (7.0)	
Crohn's disease	8 (2.3)	3 (4.2)	5 (1.8)	
Radiation enteritis	21 (6.1)	4 (5.6)	17 (6.2)	
Others	5 (1.4)	2 (2.8)	3 (1.1)	

*Values were presented as n (%)*.

*SBS, short bowel syndrome*.

Overall, 64 (18.6%) patients had end-jejunostomy with no colon-in-continuity, 124 (35.9%) had jejuno-colonic anastomosis with partial colon-in-continuity, and 157 (45.5%) had jejuno-ileal anastomosis with an intact colon. The mean residual small intestine length was 80.8 ± 45.6 cm, and the median length was 80 cm (range, 50–104 cm). In total, 247 (71.6%) patients had the main remnant jejunum, and 256 (74.2%) patients had colon integrity (Table 3).

**Table 3:** The intestinal anatomy of adult patients with SBS with and without cholelithiasis.

**Variables**	**All**	**Cholelithiasis**	**Non cholelithiasis**	***p* value**
**Number of patients**	345	72	273	NA
Anatomy type				0.758
Jejunostomy (type I)	64 (18.6)	14 (19.4)	50 (18.3)	
Jejunocolic anastomosis (type II)	124 (35.9)	28 (38.9)	96 (35.2)	
Jejunoileal anastomosis (type III)	157 (45.5)	30 (41.7)	127 (46.5)	
Remaining small intestine length (cm)				0.628
Mean	80.8 ± 45.6	82.7 ± 46.1	80.2 ± 45.5	
Median	80 (50–104)	80 (56–108)	80 (50–104)	
Remaining small intestine type				**0.013**
Jejunum predominantly	247 (71.6)	60 (83.3)	186 (68.1)	
Ileum predominantly	98 (28.4)	12 (16.7)	87 (31.9)	
Ileocecal valve intact	165 (47.8)	33 (45.8)	132 (48.4)	0.704
Colon in continuity	281 (81.4)	58 (80.6)	223 (81.7)	0.826
Colon integrity	256 (74.2)	51 (70.8)	205 (75.1)	0.463

*Values were presented as n (%), or mean ± SD, or median (first-to-third interquartile range)*.

*p <0.05 is indicated by black bold*.

*SBS, short bowel syndrome*.

### Prevalence of Cholelithiasis

**Figure 2:**
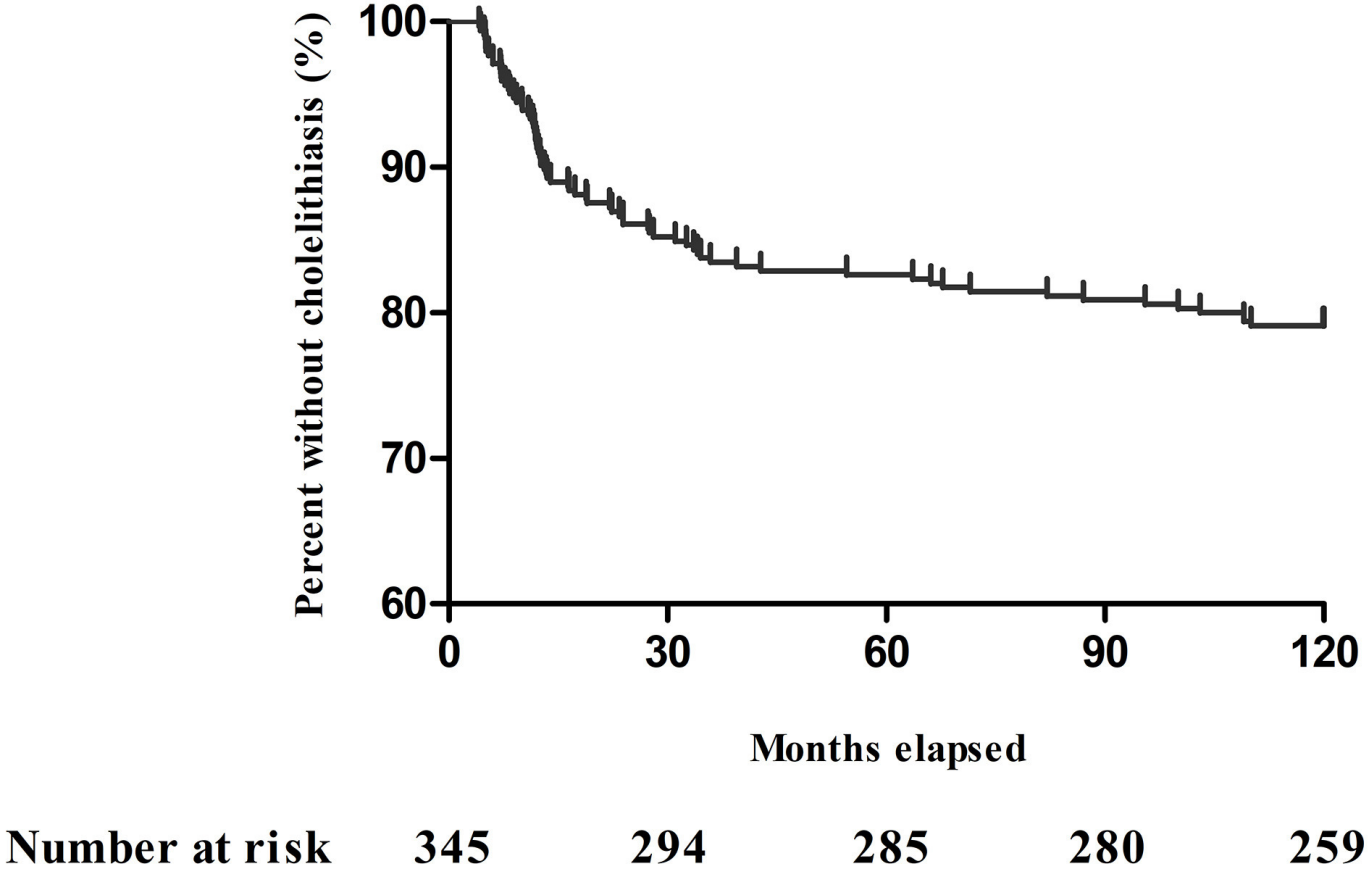
Kaplan–Meier plot showing adult patients with short bowel syndrome without cholelithiasis during the 10-year period.

According to the Kaplan–Meier analysis, the incidence of cholelithiasis was 20.9% (72/345) over an observation period of 10 years; the remaining 273 (79.1%) patients had no cholelithiasis (Figure 2). The features of the entire study population stratified by the abdominal imaging diagnosis are detailed in Tables 1–3. Cholelithiasis was present in 14 (19.4%) patients with end-jejunostomy without colon-in-continuity and 58 (80.6%) with jejuno-colonic or jejuno-ileal anastomosis and colon-in-continuity (Table 3).

Patients were divided according to the occurrence of cholelithiasis into the cholelithiasis group (*n* = 72) and the non-cholelithiasis group (*n* = 273). Sex, the PG-SGA grade, and PN dependence were significantly different in SBS patients with cholelithiasis compared with SBS patients without cholelithiasis (*p* < 0.05) (Table 1). However, the two groups were similar in age, height, body weight, the BMI, NRS-2002 score, presence of hypertension, presence of diabetes mellitus, and levels of hemoglobin, albumin, and other indicators (all *p* > 0.05, Table 1). The cholelithiasis group had a greater incidence of remnant jejunum than the non-cholelithiasis group (60 [83.3%] vs. 186 [68.1%], *p* < 0.01) (Table 3), but there were no significant differences between the two groups in terms of the primary disease, type of anatomy, length of the remnant small intestine, presence of an intact ileocecal valve, colon-in-continuity, and colon integrity (all *p* > 0.05, Tables 2, 3).

### Risk Factors for Cholelithiasis

According to Cox proportional hazard model analyses, the independent predictors for cholelithiasis in the overall population of patients with SBS are shown in Table 4. Only two variables, the type of remaining small intestine (mainly the remnant jejunum) and PN dependence, were found to have significant predictive effects. Hazard ratio (HR) for the two independent predictors of cholelithiasis were 2.163 (95% CI: 1.156–4.047, *p* = 0.016) and 1.783 (95% CI: 1.077–2.952, *p* = 0.025), respectively (Table 4 and Supplementary Figure 3).

**Table 4:** The Cox proportional hazard model analysis of the independent variables associated with cholelithiasis in adult with SBS.

**Independent variable**	***p* value**	**Hazard ratio**	**95% CI for exp(B)**
Age (years)			
<65/≥65	0.562	0.844	0.475–1.498
Sex			
Male/Female	0.062	1.572	0.977–2.528
Remaining small intestine (cm)			
>100/ ≤ 100	0.113	1.698	0.882–3.267
Ileocecal valve intact			
Yes/No	0.802	0.934	0.546–1.596
Colon integrity			
Yes/No	0.351	1.321	0.736–2.371
Colon in continuity			
Yes/No	0.898	0.961	0.525–1.760
Remaining small intestine type			
Mainly remnant ileum/jejunum	**0.016**	2.163	1.156–4.047
PN dependence			
No/Yes	**0.025**	1.783	1.077–2.952

*p < 0.05 is indicated by black bold*.

*SBS, short bowel syndrome; HR, Hazard ratio; CI, confidence interval*.

### Clinical Consequences of Cholelithiasis

Of the 72 patients in the cholelithiasis group, 44 were asymptomatic, with cholelithiasis found incidentally on abdominal imaging. Twenty-eight patients with cholelithiasis developed symptoms and/or complications, including 23 with acute cholecystitis and cholangitis and five with acute pancreatitis (Table 5). Proportions of acute cholecystitis or cholangitis and acute pancreatitis were significantly increased in the cholelithiasis group compared with the non-cholelithiasis group (31.9 vs. 7.7%, *p* < 0.01 and 6.9 vs. 1.1%, *p* = 0.003, respectively; Table 5). Of the 28 patients with symptomatic cholelithiasis, 13 were treated conservatively, while 15 required surgical intervention. The proportion of cholecystectomy or endoscopic sphincterotomy was significantly increased in the cholelithiasis group compared with the non-cholelithiasis group (19.4 vs. 4.0%, *p* < 0.01, Table 5).

**Table 5:** The complication in adult patients with SBS with and without cholelithiasis.

**Variables**	**All**	**Cholelithiasis**	**Non cholelithiasis**	***p* value**
**Number of patients**	345	72	273	NA
**Biliary complication**				
Acute cholecystitis or cholangitis (%)	44 (12.8)	23 (31.9)	21 (7.7)	**<0.001**
Acute pancreatitis (%)	8 (2.3)	5 (6.9)	3 (1.1)	**0.003**
**Conservative therapy**	20(5.8)	13(18.1)	7(2.6)	**<0.001**
**Surgical therapy**				
Cholecystectomy or ES (%)	25 (7.2)	14 (19.4)	11 (4.0)	**<0.001**
PTGD (%)	7(2.0)	1 (1.4)	6 (2.2)	0.665

*Values were presented as n (%)*.

*P < 0.05 is indicated by black bold*.

*SBS, short bowel syndrome; ES, endoscopic sphincterotomy; PTGD, percutaneous transhepatic gallbladder drainage*.

## Discussion

To our knowledge, this is the largest study to specifically explore the incidence, risk factors, and clinical consequences of cholelithiasis in a longitudinal cohort of adults with SBS. The incidence of cholelithiasis in adults with SBS was 20.9% during the 10-year observation period, and the median duration from SBS diagnosis to the development of cholelithiasis was approximately 15 months. The independent risk factors for cholelithiasis in adults with SBS were the type of remaining small intestine (mainly the remnant jejunum) and PN dependence. Moreover, cholelithiasis was associated with adverse clinical consequences in some patients with SBS.

Individuals with SBS are susceptible to cholelithiasis, which can result in adverse clinical consequences. Our study found that the incidence rate of cholelithiasis was 20.9% in adults with SBS. Previous studies with smaller populations (119 patients, 109 patients, 81 patients, and 71 patients) found the following prevalence rates of cholelithiasis: 6.2–47% in patients with chronic intestinal failure (CIF) receiving HPN (4, 14, 15, 19), >5–20% in patients with cholelithiasis in the non-HPN Asian general adult population, and 10–20% in the European and United States adult population (1, 20). Our results expand on those of previous reports regarding the prevalence and incidence of cholelithiasis in CIF. These earlier studies were undertaken during an era when intravenous hyperalimentation was common practice, the studies were usually cross-sectional in nature, and consequently and most importantly, some studies did not exclude patients with pre-existing cholelithiasis. This may explain why the reported incidence of cholelithiasis has been reported to be as high as 23–100% over a mean duration of PN of only 14–39 months in these earlier studies (3, 19, 21). In contrast, we followed-up with patients longitudinally for much longer in the present study and their intravenous regimens were consistent with current ESPEN guidelines on CIF in adults, including a reduction in the amount of soybean-oil-based lipid and enteral feeding (22). Therefore, the present data may represent a more accurate assessment of the likelihood that a patient with SBS will develop cholelithiasis during the course of their treatment and require intervention for cholelithiasis-related morbidity; therefore, it might be used as the basis for formulating future treatment policies.

The risk factors predisposing patients to cholelithiasis formation include obesity, diabetes mellitus, estrogen and pregnancy, hemolytic diseases, and cirrhosis in the general population (20). In our study, the type of remaining small intestine (mainly the remnant jejunum) and PN dependence were the independent risk factors significantly associated with the incidence of cholelithiasis in adults with SBS. This finding is consistent with those of previous studies (19, 23, 24), which strongly suggest that patients who receive long-term PN are at an increased risk for the development of cholelithiasis. It is of interest to consider if other conditions (such as ileal disorders) can contribute to the development of cholelithiasis, independent of a coexisting factor (i.e., fasting periods associated with ileal disease). It has been suggested that ileal disorders (including Crohn disease), a previous ileal resection, or both, increase the risk of cholelithiasis, with a prevalence ranging from 17 to 34% (4). A retrospective series of 35 patients with gallbladder *in situ* and SBS (20 with PN, 15 without PN) reported 10 cases (28.6%) of cholelithiasis, all of which were symptomatic and required cholecystectomy (25). According to previous studies, patients with SBS are at an increased risk for developing cholelithiasis if the intestinal remnant is <120 cm, terminal ileum is resected, and PN is long term (4, 25). Our study results are partly consistent with those of previous studies. The incidence of cholelithiasis may be higher in the resection of the ileocecal valve and remnant jejunum in patients with SBS. We speculate that the possible main reason for this may be that normal entero-hepatic circulation is damaged, which aggravates cholestasis and promotes the formation of cholelithiasis.

Previous studies have reported that more than one-third of patients with SBS develop cholelithiasis during the course of their disease, usually within the first 2 years after resection (3, 4, 21, 25, 26). Our findings indicate that the median duration from SBS diagnosis to the development of cholelithiasis is approximately 15 months, which is consistent with findings of previous studies. The pathophysiology of cholelithiasis in patients with SBS is multifactorial and varies depending on the underlying disease and bowel anatomy. However, the pathogenesis of cholelithiasis in patients with SBS is not completely understood. At least two factors may contribute to its pathogenesis: an altered bile composition due to the enterohepatic circulation of bile acids impaired by ileal resection results in a reduction in hepatic bile acid secretion, which becomes supersaturated with cholesterol (18); and bile stasis due to the diminished enteric hormonal stimulation of gallbladder contractions that may lead to biliary stasis and biliary sludge formation (18). During prolonged starvation, there is modification of normal bile acidification and a reduction in the secretion of cholecystokinin (27). Gallbladder contractility is consequently reduced, promoting biliary stasis and the formation of biliary sludge (28). Interruption of the enterohepatic circulation and loss of bile salts following ileal resection leads to cholesterol supersaturation and sludge formation (29). Current recommendations for the prevention of cholelithiasis in IF include allowing oral nutrition as tolerated and limiting use of narcotics and anticholinergics (22).

In the current study, 61.2% of patients with cholelithiasis were asymptomatic, while 38.8% were symptomatic, which is significantly lower than 77.4% of long-term home parenteral nutrition patients with cholelithiasis were symptomatic that reported in previous study (26). Of the 28 patients with symptomatic cholelithiasis, 20.8% required surgical intervention, which is a slightly higher proportion than that reported in previous study (26). Moreover, proportions of biliary complications (including acute cholecystitis or cholangitis and acute pancreatitis) were significantly higher in the cholelithiasis group than in the non-cholelithiasis group. Our study results suggest that cholelithiasis is associated with adverse clinical outcomes. Therefore, cholelithiasis should be closely monitored in patients with SBS.

The present study's results have implications in the role of surgical intervention (including cholecystectomy, endoscopic sphincterotomy, and percutaneous transhepatic gallbladder drainage) in SBS, especially in patients with symptomatic cholelithiasis. Although previous studies uniformly recommended prophylactic cholecystectomy (3, 4, 21, 25), current recommendations from ESPEN have taken an opposing view, advising that intervention be reserved for symptomatic or complicated gallstone disease alone (22). After first addressing the role of prophylactic surgery in any patient with SBS and an apparently healthy gallbladder, the current data show that although a significant proportion of patients with SBS will eventually develop gallstones, the majority will not. Furthermore, when intervention was required to manage cholelithiasis, our data suggested that this was safe, and the high morbidity and mortality reported by early studies were not observed. However, our results showed that once cholelithiasis develops, some patients with SBS, unlike individuals without cholelithiasis, will experience symptoms or complications, and the majority will need intervention. Therefore, the practice of routinely undertaking prophylactic cholecystectomy in patients with SBS without cholelithiasis is not supported by our findings.

Although the present study is based on a clinical audit database prospectively maintained at Jinling Hospital's Intestinal Failure Clinical Nutrition Center over several decades, it has several significant limitations. First, this was a retrospective, single-institution study performed at a tertiary hospital center. Second, there were no data regarding the composition of cholelithiasis. Third, as most patients with SBS from our Intestinal Failure Clinical Nutrition Center were adults, we only evaluated the incidence and risk factors for cholelithiasis in adults with SBS. The prevalence and risk factors for cholelithiasis in pediatric patients remain unclear. Fourth, the absolute risk of cholelithiasis attributable to SBS remains unknown due to the lack of a normal control group of patients without SBS. Lastly, even though the detailed and regular follow-up of a large cohort of patients receiving long-term parenteral support was a key strength of the present study, the patients did not undergo formal planned surveillance with regular ultrasonography, only cross-sectional imaging or ultrasonography as required for clinical reasons. Although the percentage of patients who developed cholelithiasis in the present study was high, our figures might still represent an underestimation. Therefore, large, multicenter, prospective studies focusing on the formation of cholelithiasis in both pediatric and adult patients with SBS should be conducted in the future.

## Conclusions

Adult patients with SBS are at a particularly high risk of developing cholelithiasis. The independent predictors of cholelithiasis were type of remaining small intestine (mainly the remnant jejunum) and PN dependence. Cholelithiasis can lead to adverse clinical consequences that should be closely monitored and require prophylactic intervention.

## Data Availability Statement

The original contributions presented in the study are included in the article/Supplementary Material, further inquiries can be directed to the corresponding author.

## Ethics Statement

The studies involving human participants were reviewed and approved by the Research Ethics Committee of the Jinling Hospital. The ethics committee waived the requirement of written informed consent for participation.

## Author Contributions

XW and XG equally contributed to the conception and design of the study. LZ and SW contributed to the design of the study. SW and YX contributed to the acquisition and analysis of the data. YX, DZ, and DS contributed to the analysis of the data. XG, LZ, and DS contributed to the acquisition, analysis, and interpretation of the data. All authors drafted the manuscript, critically revised the manuscript, agreed to be fully accountable for ensuring the integrity and accuracy of the work, and read and approved the final manuscript.

## Funding

This study was supported by the National Natural Science Foundation of China (81470797, 81770531), the Science Foundation of Outstanding Youth in Jiangsu Province (BK20170009), the National Science and Technology Research Funding for Public Welfare Medical Projects (201502022), and Military Medical Innovation Project (18CXZ031).

## Conflict of Interest

The authors declare that the research was conducted in the absence of any commercial or financial relationships that could be construed as a potential conflict of interest.

## Publisher's Note

All claims expressed in this article are solely those of the authors and do not necessarily represent those of their affiliated organizations, or those of the publisher, the editors and the reviewers. Any product that may be evaluated in this article, or claim that may be made by its manufacturer, is not guaranteed or endorsed by the publisher.
