# Supplementary material for: Prevalence, Risk Factors, and Complications of Cholelithiasis in Adults With Short Bowel Syndrome: A Longitudinal Cohort Study
Source: Front Nutr. 2021 Nov 29;8:762240. doi: 10.3389/fnut.2021.762240 (PMC8667726; doi:10.3389/fnut.2021.762240)
Supplement: Supplementary file 1 [file Table_1.DOCX]

**Table S1. Univariate and multivariate analysis of the independent variables associated with cholelithiasis in the entire population of patients with SBS study**

| **Independent variable** | **Univariable analysis** | | **Multivariable analysis** | |
| --- | --- | --- | --- | --- |
|  | **OR (95% CI)** | ***p* value** | **OR (95% CI)** | ***p* value** |
| Age (years)  <65/≥65 | 1.146  (0.601–2.1831) | 0.680 |  |  |
| Sex  Male/Female | 1.627  (0.950–2.787) | 0.076 |  |  |
| Diabetes mellitus  No/Yes | 0.686  (0.147–3.141) | 0.622 |  |  |
| Hypertension  No/Yes | 1.187  (0.571–2.467) | 0.646 |  |  |
| Tumor  No/Yes | 1.380  (0.778–2.447) | 0.271 |  |  |
| Remaining small intestine (cm)  >100/≤100 | 1.491  (1.066–2.085) | **0.020** | 1.356  (0.951–1.934) | 0.092 |
| Ileocecal valve intact  Yes/No | 0.904  (0.537–1.522) | 0.704 |  |  |
| Colon integrity  Yes/No | 0.806  (0.452–1.435) | 0.463 |  |  |
| Colon in continuity  Yes/No | 0.929  (0.480–1.796) | 0.826 |  |  |
| Remaining small intestine type  Mainly remnant ileum/jejunum | 2.299  (1.176–4.495) | **0.015** | 2.333  (1.181–4.590) | **0.015** |
| PN dependence  No/Yes | 2.073  (1.220–3.523) | **0.007** | 1.790  (1.019–3.142) | **0.043** |

*p* value <0.05 is indicated by black bold.

SBS, short bowel syndrome; OR, odds ratio; CI, confidence interval.
