# Supplementary figures and images for: Prevalence, Risk Factors, and Complications of Cholelithiasis in Adults With Short Bowel Syndrome: A Longitudinal Cohort Study
Source: Front Nutr. 2021 Nov 29;8:762240. doi: 10.3389/fnut.2021.762240 (PMC8667726; doi:10.3389/fnut.2021.762240)

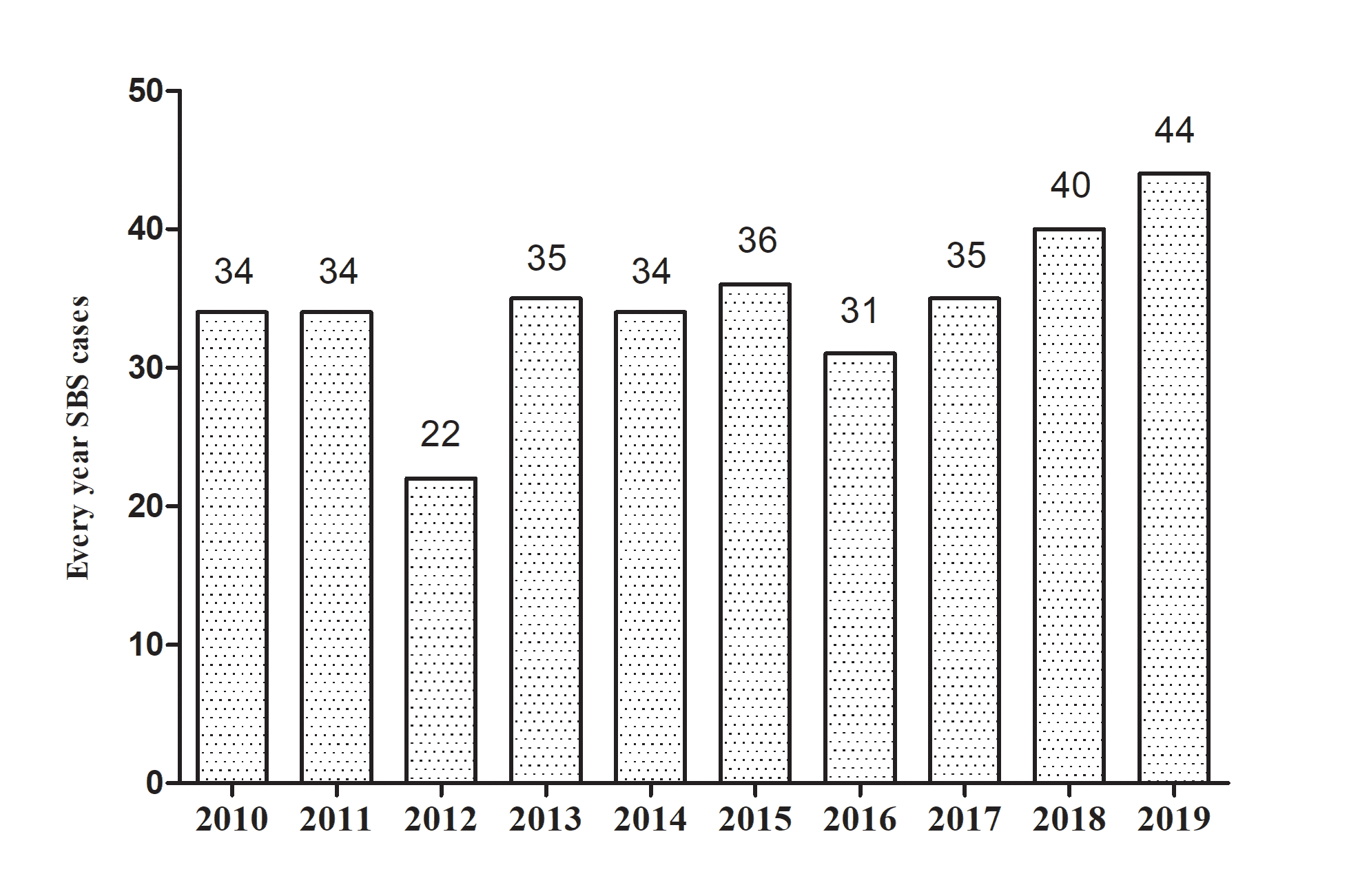

Supplement: Supplementary file 2 [file Image_1.TIF]

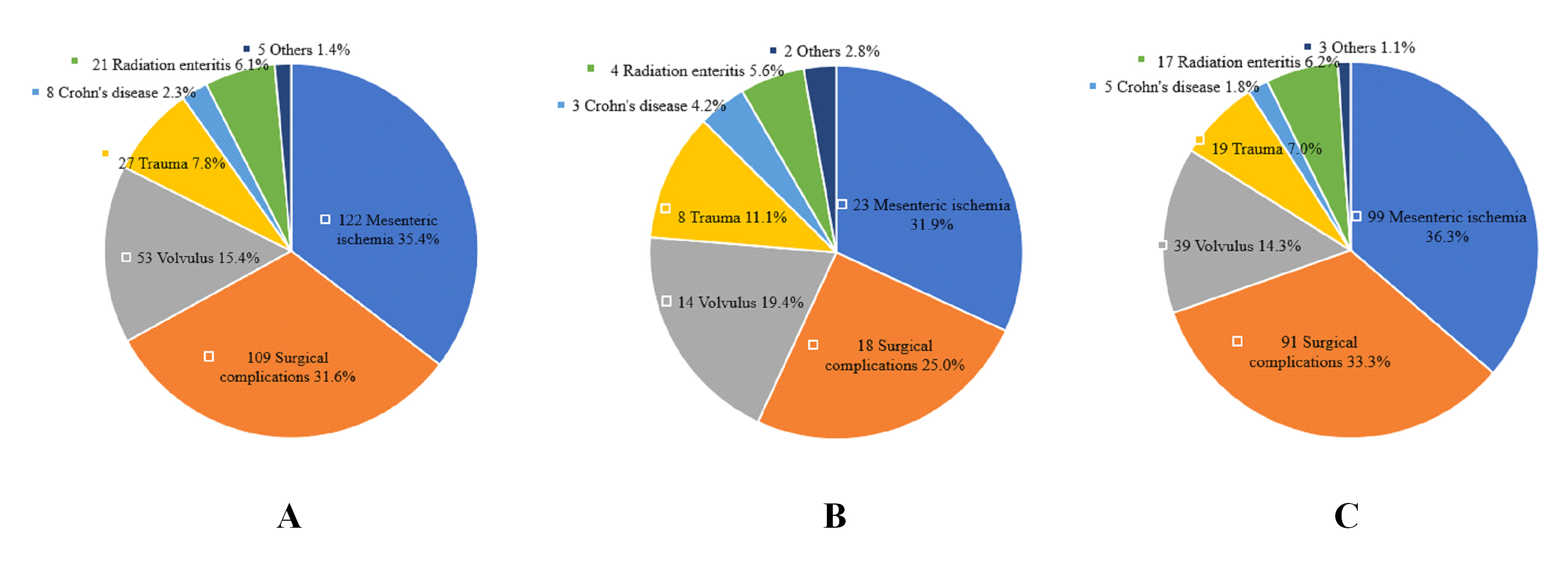

Supplement: Supplementary file 3 [file Image_2.JPEG]

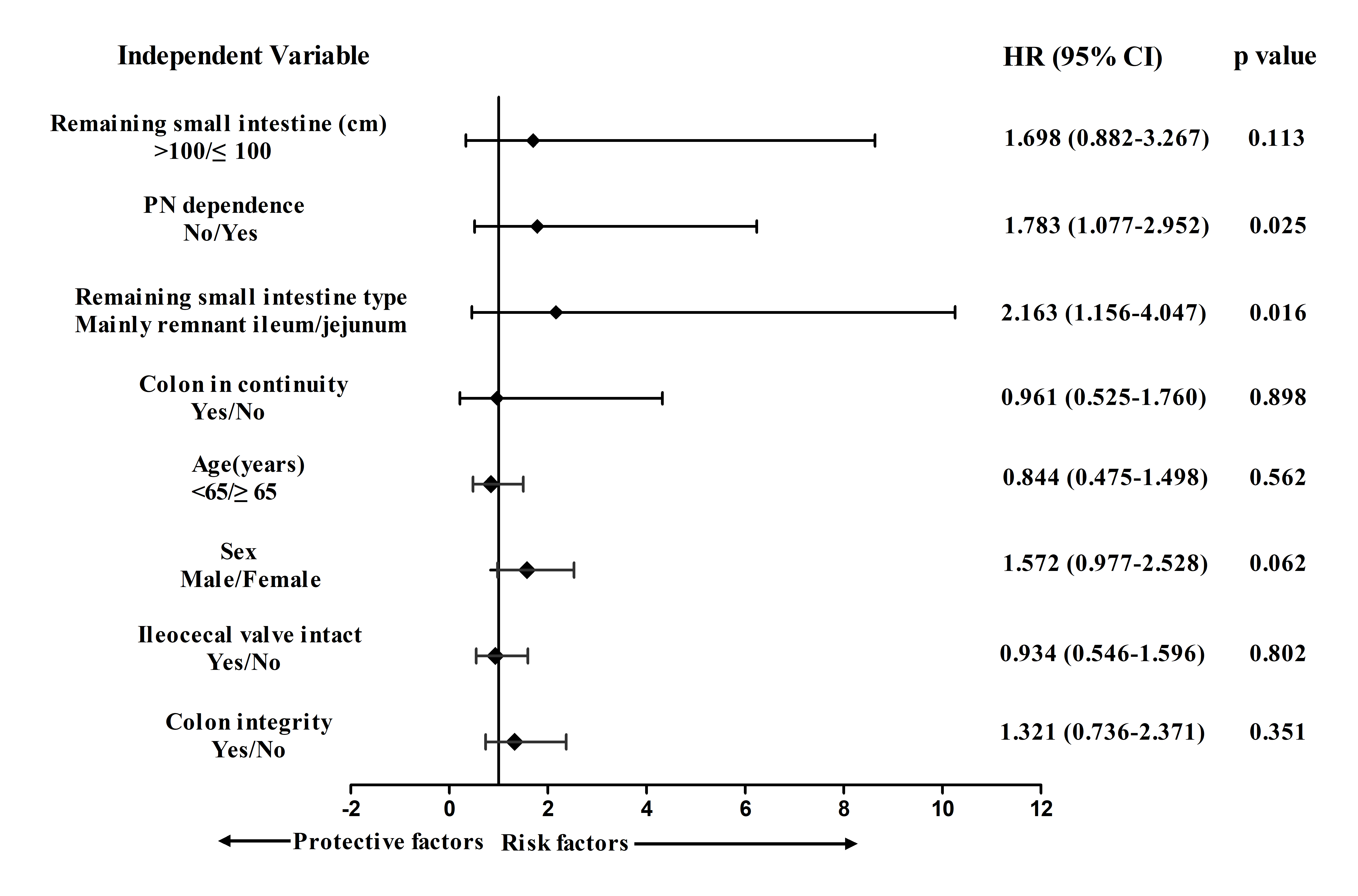

Supplement: Supplementary file 4 [file Image_3.JPEG]
